# Computational Tools for Interpreting Ion Channel pH-Dependence

**DOI:** 10.1371/journal.pone.0125293

**Published:** 2015-04-27

**Authors:** Ivan Sazanavets, Jim Warwicker

**Affiliations:** Manchester Institute of Biotechnology, The University of Manchester, 131 Princess Street, Manchester, M1 7DN, United Kingdom; Xuzhou Medical College, CHINA

## Abstract

Activity in many biological systems is mediated by pH, involving proton titratable groups with pKas in the relevant pH range. Experimental analysis of pH-dependence in proteins focusses on particular sidechains, often with mutagenesis of histidine, due to its pKa near to neutral pH. The key question for algorithms that predict pKas is whether they are sufficiently accurate to effectively narrow the search for molecular determinants of pH-dependence. Through analysis of inwardly rectifying potassium (Kir) channels and acid-sensing ion channels (ASICs), mutational effects on pH-dependence are probed, distinguishing between groups described as pH-coupled or pH-sensor. Whereas mutation can lead to a shift in transition pH between open and closed forms for either type of group, only for pH-sensor groups does mutation modulate the amplitude of the transition. It is shown that a hybrid Finite Difference Poisson-Boltzmann (FDPB) – Debye-Hückel continuum electrostatic model can filter mutation candidates, providing enrichment for key pH-coupled and pH-sensor residues in both ASICs and Kir channels, in comparison with application of FDPB alone.

## Introduction

The dependence on pH of biological systems plays a key role in their structure and function [1]. Both normal and pathological processes are regulated by changes in the intracellular pH [2,3] and extracellular pH [4,5]. Many energy transduction mechanisms of cells depend on proton-coupled electron transport and proton gradients [6]. Physiological pH is tightly regulated and small changes from 0.1 to 0.3 pH units can lead to dramatic changes in behaviour [2,6]. Our molecular understanding of how pH changes are used in nature to mediate function is increasing, for example in the salvage mechanism of antibodies [7], and more generally in protein-protein [8,9], and ligand-receptor interactions [10,11].

A theoretical analysis of pH-dependence typically involves a structure-based application of continuum electrostatics models, often based on numerical solution of the Poisson-Boltzmann (PB) equation [12,13]. Two productive areas for linking theory and experiment have been protein folded state stability and catalysis. Each of these cases has their own problems. In calculating folding state stability, conformational characterisation of the unfolded state is difficult. Generally it is assumed that charge interactions in the folded state dominate the pH-dependence [14]. With catalysis, the biggest problem lies in predicting the delicate balance of dehydration and protein solvation in a relatively buried active site [15]. For ion channels transitioning between open and closed forms, there are questions of both conformational variability and the balance of energetics terms. Ideally a calculation will be differencing between structural models for open and closed forms, but these may not be available. Additionally, it is not well understood at this point whether pH-sensor groups tend generally to be solvent accessible or solvent inaccessible, or indeed switch between these states. Rationalising and predicting pH-dependence in ion channels is therefore likely to be a challenging task. Since it is thought that almost all ion channels are regulated by pH to some degree [16], it is worth assessing our current ability in this field. This report uses two extensively studied systems to investigate this area, acid-sensing ion channels (ASICs) and inwardly-rectifying potassium (Kir) channels. These systems have previously been mutagenised to probe pH-dependence.

Kir ion channels are the main regulators of K^+^ homeostasis in the body [17]. Kir channel activity controls many different processes, such as heart rate, vascular tone, insulin secretion and salt and fluid balance [18]. Kir channels are inhibited by intracellular acidity [17]. Whilst all channels in the Kir superfamily are sensitive to some extent to intracellular pH and are inhibited by low pH of ~5.0 [3], only a small subset are inhibited by pH in the physiological range. This subset consists of Kir1.1, Kir4.1, and Kir4.2 and also includes heteromeric Kir4.x/5.1 channels [3]. Some other members of the Kir superfamily are modulated by pH without the characteristic inhibition shown by channels labelled as pH-sensitive. Kir2.3 is activated by both intracellular and extracellular alkalisation, and the activity of Kir2.4 is enhanced with intracellular alkalisation, whilst Kir7.1 has maximum activity at pH 7.0 [18]. Work aimed at deciphering the molecular basis of pH-dependence in Kir channels has been reviewed [3]. In brief, K80 (rat Kir1.1) was established early on as an important residue [19], with the threonine equivalent to T70 in Kir1.1 also modulating pH-dependence [20]. However, other factors also play a role. Mutation of K80 significantly shifts, rather than removes, the midpoint of pH-dependence [21]. A Kir1.1 orthologue from puffer fish does not have this lysine but exhibits pH sensitivity [22]. In addition, the bundle crossing is coupled to pH gating [23].

As a counterpoint to Kir channels, ASICs open in response to acidic extracellular pH [24]. These proton-gated sodium ion channels are members of the degenerin/epithelial sodium channel family [25] and are found throughout mammalian central and peripheral nervous systems [26]. ASICs are involved in a number of neuronal processes, such as modulation of synaptic transmission [27], detection and processing of sensory information [28], nociception and pain detection [29], memory formation and the expression of fear [30]. Gating properties and pH-sensitivity, measured by the pH_50_ for ion channel opening (i.e. the pH of half-maximal activation), vary between different types of ASICs. For mammalian ASICs, pH sensitivity has been recorded [31] as follows: ASIC1a, ASIC1b and ASIC3 are pH sensitive. ASIC2a has a low extracellular acid sensitivity. ASIC2b and ASIC4 are not known to be acid sensitive.

Calcium ions modulate the pH dependent gating of ASICs. It has been demonstrated that calcium and protons compete for binding to the channel, with calcium binding being favoured in the closed state, while proton binding favours the open or desensitised state [31], with calcium ions needing to be displaced before a channel can open [32]. The apparent pH sensitivity of mammalian ASIC1 and ASIC3 is reduced with increased extracellular Ca^2+^ concentration [33]. Calcium-binding sites are believed to be present in ASICs in two distinct groups; one type that predominantly determines the pore block and another type that predominantly modulates calcium regulation of pH sensitive gating [34,35]. E426 and D433 have been identified as crucial for calcium block of ASICs [34]. Calcium can also accelerate the recovery from desensitization of ASICs [26].

Proton sensitive ASICs are known to reside in at least three different states: a resting state, where channels are closed to the passage of ions but can be activated by a drop in extracellular pH; an open conducting state; and a desensitised state (also known as steady-state inactivation (SSIN) [5], where a channel is closed and insensitive to extracellular acidification [36]. Recovery from the desensitised state requires prolonged exposure to an alkaline pH [5].

Computational prediction methods use structures deposited in the Protein Data Bank (PDB) [37] of the biomolecules being investigated or comparative models constructed from related structures. Although considerable progress in membrane protein structure solution has been made in recent years [38], it is often the case that available conformations will not capture the functional range (e.g. open, closed, desensitised ion channels). The first crystal structure of an ASIC was reported in 2007 [26], and represents the closed, desensitised state. Further ASIC structures [39] were also solved in the desensitised conformation, but with variation in the orientation of transmembrane segments. More recently, crystal structures of ASIC have been obtained including an ion channel in complex with psalmotoxin-1 [25,40], with a conformation that is interpreted as possessing a partially open channel [40].

It is generally believed that only a limited number of functional pH sensing residues are responsible for pH sensitivity in ion channels. These are sought through site directed mutagenesis studies, often replacing a candidate pH sensor with a structurally related but functionally neutral residue, or with alanine. The effect on pH dependence is measured (e.g. through pH_50_ of activation), and a potential role of the candidate residue assessed. Various methods are employed for identifying candidate functional residues (CFRs). CFRs are commonly predicted through sequence level comparison of ion channels differing in pH sensitivity. For example, all Asp, Glu and His residues that are present in pH sensitive ASIC2a but are not in the pH insensitive splice variant ASIC2b have been chosen as CFRs [41], and also groups that differ in sequence between rat ASIC1a (pH sensitive) and lamprey ASIC1 (pH insensitive) were considered as CFRs [42]. A similar strategy has been used in a number of studies where mutation chimeras, based on differences in the sequences between ion channels of varying pH sensitivity, were created. Segments of sequence that differ between mildly pH sensitive ASIC2a and a much more pH sensitive ASIC1a have been identified and examined as CFRs, through synthesising chimeras [35]. In other work, CFRs have been identified through examining differences in sequences of ASIC1 from different species, both pH sensitive and pH insensitive [43], and from differences between the sequences of zebrafish ASIC4.1 (pH sensitive) and ASIC4.2 (pH insensitive) [44]. In a variation of this strategy, CFRs were generated from ionisable residues conserved between pH sensitive ASIC1a and ASIC3 channels [32].

CFRs are also determined from examination of ion channel crystal structures, looking for groups that appear interesting through their location, orientation or proximity. Within the crystal structure of ASIC1a a negatively charged pocket in the channel, consisting of three pairs of acidic groups, was suggested to play a role in acid sensing [26]. A pair of aromatic residues at the junction of the extracellular and transmembrane domains has been implicated in the gating function of ASICs [45]. Several studies have considered residues within and preceding the second transmembrane region of ASIC as CFRs [34,36,46].

Computational methods are also used to identify CFRs. Mutagenesis has been directed at Asp, Glu, and His with predicted pKa values in the physiological pH range (between pH 5 and 8), where these values were estimated with Poisson-Boltzmann methods [5]. Protonation probability of four acidic pairs (three in the acidic pocket and one other) has been predicted for ASIC1a [47]. Molecular dynamics of ASICs has been used to suggest CFRs [48]. Whilst computational methods can use less resource than experimental methods, the resulting predictions need to be assessed for their effectiveness. Additionally, the most effective computational methods are not yet clear. The current work addresses these issues using Finite Difference Poisson-Boltzmann (FDPB) calculations [12,13] and the derivative Finite Difference Poisson-Boltzmann/Debye-Hückel (FD/DH) method [49]. Typically FDPB methods, based on a single conformation, generally over-estimate pKa changes from the unfolded to folded states (∆pKas), since conformational relaxation through a proton titration is absent. FDPB methods with the large dielectric differential between protein and solvent are susceptible to this problem. In FD/DH, a FDPB calculation is combined with a DH calculation [14] for ionisable groups above a threshold solvent accessibility. The DH calculation is computed purely for charge interactions in water (and with counterions), so that the effects of a protein-solvent boundary are removed. Importantly, for groups that are buried from water, in the FD/DH method, the boundary dominates. The result is that buried groups can be associated with larger ∆pKas, whilst water-accessible groups tend to have smaller ∆pKas. Overall this leads to better agreement with experimental data than does FDPB on a single conformation [49]. The FD/DH method is an alternative to FDPB combined with the sampling of multiple conformations (e.g. the multiconformation continuum electrostatics method, MCCE, [50]). Transposed to the prediction of CFRs for acid sensing, it is anticipated that FD/DH will generate a smaller list of residues than will FDPB. It is particularly effective in delineating the properties of buried groups [51], is fast and can therefore be easily applied to multiple targets (e.g. ASIC isoforms and paralogues). This is the starting point for our work, comparing for example with the application of FDPB methods for CFR identification in ASICs [5].

A key issue brought out in the current work is the relationship between pH-sensing residues and pH-coupling residues. The former are responsible for pH-dependent switches and will have ionisation state changes between conformations, over the pH-range in question, whilst the latter couple to pH-dependence but do not change ionisation state in the relevant range. Both types have residues that can alter observed pH-dependence (e.g. mid-point for a pH-induced transition), and are thus difficult to distinguish experimentally.

## Materials and Methods

### Structures and Comparative Models

The original chicken ASIC1a structure (PDB id 2qts) represents a closed, presumed desensitised form [26]. Subsequent structures, with psalmotoxin at different pH values (4fz0 pH5.5; 4fz1 pH 7.25), give an expanded pore and are presumed to give insight into the open channel [40]. For the current study 2qts is used to model the closed form and 4fz0 (with a larger pore opening than 4fz1) as indicative of the open form. The entire trimer structures are used in calculations, with the caveat that all monomers are trimmed to the minimum common length over the structures studied, to facilitate differential analysis. This was required since slightly different ASIC1a constructs were used when crystallising the closed desensitised and partially open (toxin bound) forms. Trimming was performed through analysis of the common regions that are structurally resolved following superposition of ASIC1a crystal structures in Swiss PDB Viewer [52].Thus the monomers range from C50 to L450 (chicken ASIC1a numbering). Trimming affects the intracellular amino and carboxy termini of the transmembrane helices, on the opposite side of the membrane from the extracellular sites of pH-sensing.

Where comparative models are required, in the absence of experimental structures, the sequence to be modelled is aligned with a target structure using Clustal [53], and the sidechains repacked for the model. Mainchain geometry in comparative models is unchanged from the target structure, and sidechains re-arranged to optimise packing using an adaptation [54] of a self-consistent mean mean field method [55]. The sidechain packing model is the same as that employed for assessment of solvent exposure (upon sidechain rearrangement) in the FD/DH method [49]. For example, a model of rat ASIC4 (pH insensitive) was made from chicken ASIC1a (pH sensitive), with 52% sequence identity over the 401 amino acids of the monomer model. A model trimer is constructed from combination of individually comparative modelled monomers.

Comparative models of pH-sensitive rat Kir1.1 have been modelled based on chicken Kir2.2 in a closed form (PDB id 3spc) and a PIP_2_-bound form that initiates channel opening (3spi) [56]. These models were constructed with the same procedure as described for ASICs, but with a tetramer active form. Sequence identity between template and model was 49% over the 320 amino acids of the monomer model. Differences between calculations of pH-dependence for these two modelled Kir1.1 conformations are used to predict ionisable groups that may be involved in pH-dependent channel opening/closing.

Residues known to influence pH-dependent activities for Kir channels and ASICs were found from literature searches, referred to in part in the Introduction, and reported in the Results and Discussion section, with comparison to predictions.

### Calculations

FDPB and FD/DH calculations generally followed previous procedures [49]. Relative dielectric values of 4 for protein and 78.4 for solvent were used in FDPB and a uniform value of 78.4 for Debye-Hückel. An ionic strength of 0.15 M was included for all calculations. The procedure for defining a threshold solvent accessibility employs an assessment of maximal accessibility that can be obtained through sidechain rotamer repacking on a fixed polypeptide backbone. Sidechain repacking is accomplished with a variant [54] of a mean-field algorithm [55]. Allowed atomic contacts are determined by an overlap tolerance for van der Waals (vdW) clashes, which has been optimised to give best agreement with known pKas [49]. In benchmarking of pKa prediction methods for engineered buried ionisable groups, the FD/DH method performed well [51,57].

In some cases, and where specified, this atomic clash tolerance has been altered up and down to provide a simplistic model of conformational change around partially buried groups. The reasoning is that partially buried groups will often be those of most functional interest, and that their packing, burial, and consequently electrostatic properties, will be sensitive to relatively small perturbations in conformation. Where structures are not available for more than one conformational state, then such analysis may highlight residues in this category. This hypothesis can be tested through comparing predicted and known CFRs. The value of the optimised atomic clash parameter is 1.2 to 1.4 Å, which takes into account the use of united atoms, rather than specific non-polar hydrogens, and also empirically accounts for a degree of flexibility that is not specifically included. This parameter has been discussed previously [49]. In looking for less and more restrictive packing, these vdW tolerances have been set to 1.6 and 0.8 Å and the results differenced for ionisable groups.

For ASICs, calculations are made with trimers that have been uniformly trimmed, including the transmembrane segments. Membrane is not modelled in these calculations for eukaryotic cell membrane systems since the pH-dependent sites of interest are in the extracellular domain, away from membrane (dielectric) influence. Additionally, there is evidence that although the general non-polar surface of the transmembrane segments is clear for this overall family of proteins, the precise register (or range of registers) between protein and membrane is variable [58]. All calculated pKas are given as averages across the number of monomers contributing to the functional molecule (e.g. 3 for ASICs). Membrane is also excluded for Kir1.1 calculations, which although an approximation, again negates the need for precise placement of phospholipid molecules or a low dielectric geometrical slab. In previous computational studies of potassium channels, we found that inclusion of a membrane component does not greatly affect charge interactions close to the central pore [59], although this may not always be the case for groups determining pH-dependence.

Where calculations of pH-dependent energies are made, these relate to the ionisable group contributions to folded state stability, relative to a notional unfolded state with no interactions between ionisable groups. These are typically computed as differences between folded states (e.g. open and closed), thus removing the unfolded state term. Energetics of pH-dependence were calculated from the pKa calculations, through the overall charge on the structure [14,60]. The pKas themselves were computed with Monte Carlo sampling over ionisation states [61], following an overall scheme for separating electrostatic terms [62].

Molecules were displayed and manipulated with Swiss PDB Viewer [52] and PyMOL (The PyMOL Molecular Graphics System, Schrodinger LLC). Maps for display in PyMOL were generated (and differenced where appropriate) with in house code associated with our electrostatics analysis software.

## Results and Discussion

### Prediction of pH-sensing residues in Kir1.1

**Fig 1:**
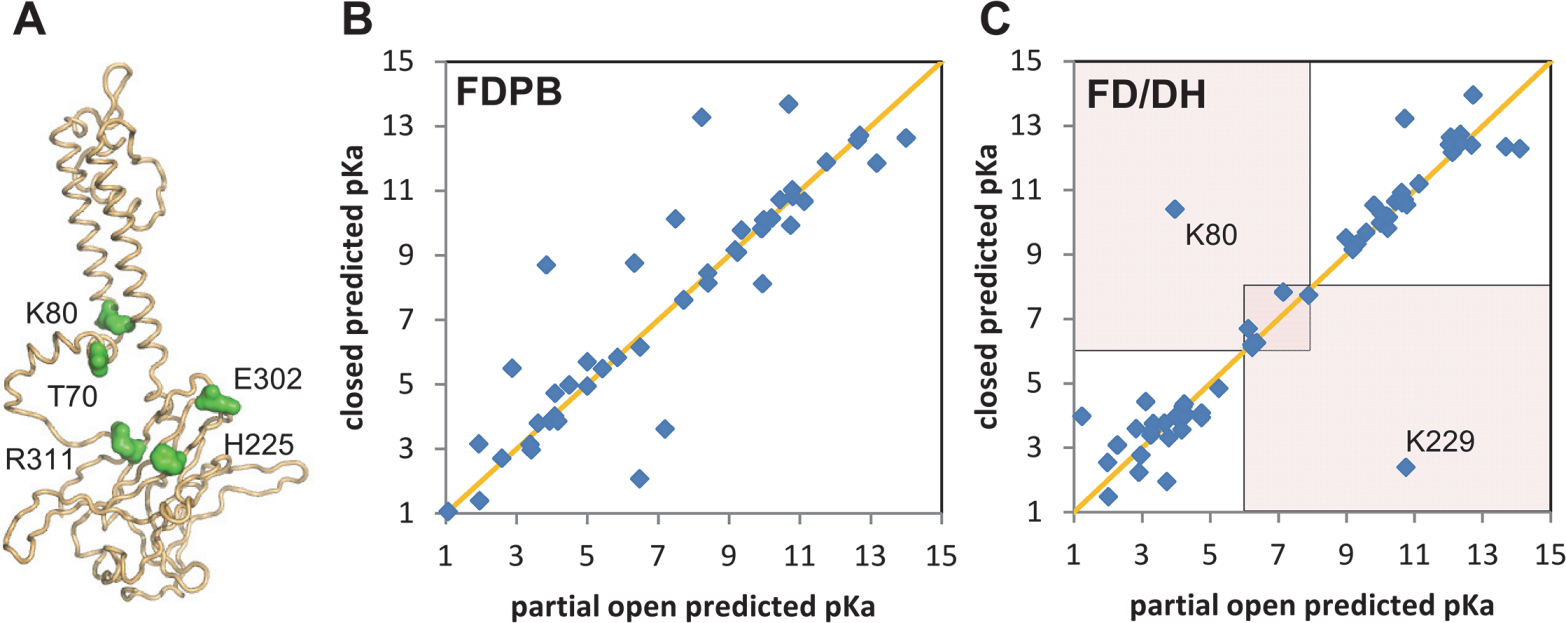
Prediction of pH-sensing residues for Kir1.1. (A) Cartoon of a Kir1.1 monomer (from the tetramer), with extracellular loops upper, intracellular domain lower and, the intervening helices marking the transmembrane regions. Residues identified as influencing the pH-dependent transition (see text) are marked with green surfaces. (B) and (C) Scatter plots of predicted pKas for closed and partially open channels, with the orange diagonal indicating equal pKas in these forms. Choosing a pH range of physiological interest between pH 6 and 8, the shaded regions (panel C) contain groups for which ionisation is predicted to change between closed and partially open forms, at physiological pH. Panel B is for FDPB calculations and panel C for FD/DH calculations, with groups K80 and K229 highlighted.

Fig 1A illustrates amino acids shown to influence pH_50_, and in principle could be true pH-sensing residues, or coupled to the pH-dependent transition. Residues that contribute directly to a pH-sensitive switch between conformations will have pKas that are different between those conformations, and that difference will overlap with the pH-range in question. In Fig 1C, this is illustrated by defining the pH-range in question as around pH 6–8, and drawing sectors that highlight predicted pKas that differ across this range, between closed Kir and partially open Kir. Thus, we are not interested in pKas that are on the diagonal (same predicted pKas in closed and partially open forms), or those that are off-diagonal but with both predictions < pH 6 or > pH 8. This strategy marks a departure from simply asking what residues have pKas around physiological pH in a single conformation. Fig 1B is computed with FDPB and Fig 1C with FD/DH. Generally, it is apparent that the spread of predictions away from the diagonal is less for FD/DH than for FDPB. This is a consequence of the feature of FD/DH in lowering the number of relatively large ∆pKa predictions, that is a problem with FDPB when the underlying conformation is not sampled or allowed to relax upon pH titration [49]. Focussing then on the off-diagonal groups in FD/DH calculations (Fig 1C) that are predicted to change protonation through physiological pH, between the closed and partially open forms, K80 and K229 are prominent.

Panels A and B of S1 Fig show predictions of pH-sensing residues, using the approximation that partially buried residues are likely to be important. Here we have taken the closed (S1A Fig) and partially open (S1B Fig) forms, and varied the packing tolerance for residues in the FD/DH model. As described in the Materials and Methods section, an optimum packing tolerance, to combine the FDPB and DH schemes, has been determined previously [49]. By differencing between calculations made with tolerances above and below this optimum value, ionisable groups are effectively being incorporated into the water-dominated DH interaction scheme more or less easily than with the optimised packing tolerance. The net effect is to identify groups that are partially buried and therefore borderline on their accessibility to the water-dominated DH scheme. We hypothesise that such groups are likely to be more changeable in electrostatic properties, with respect to relatively small conformational change, and are potential candidates for physiological pH-dependence, if the their pKas alter through the relevant pH range. Thus S1A and S1B Fig are calculated at pH 6.5, and ionisation states given as scatter plots, with ordinates from the lower and higher packing tolerance computations. Again, it is off-diagonal terms that are of most interest, i.e. those that change ionisation state. This is an empirical method, designed to give an opportunity for computation to assist predictions of CFRs where only a single conformation is available. Interestingly, K80 and K229 are again apparent, from both conformations (S1A and S1B Fig). Other substantially off-diagonal groups, again common to both conformations, are H225, E258 and D298.

These residues are potentially truely pH-sensing, contributing to the pH-switch in conformation, rather than pH-coupled, which would move the poising of a pH switch, but not actually contribute to the pH switch themselves. It is well known that K80 is an important residue [19], although it is not solely responsible [21]. The other highlighted residues are largely away from the regions of Kir 1.1 investigated with alanine scanning mutagenesis [63], but there is some evidence that H225 may be involved in pH-dependence for Kir 1.1 [64]. Revisiting the experimentally determined groups shown in Fig 1A, T70 will not appear in the current calculations since it is not ionisable (which does not preclude it coupling to the pH-dependent transition), whilst the R311-E302 salt-bridge is discussed in the next section.

### Prediction of pH-coupled residues in Kir1.1

**Fig 2:**
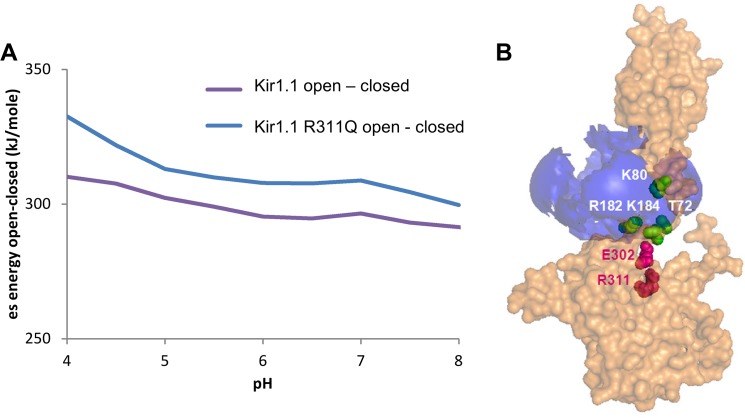
Predicted pH-sensor and pH-coupled residues in Kir1.1. (A) pH-dependence of open-closed conformation energetics predicted for rat Kir1.1 wild type and R311Q mutant. A shift in the plots, rather than a change in slope, is shown, suggesting pH-coupling rather than pH-sensing. (B) A single monomer of rat Kir1.1 is shown, but with a positive potential contour (kT/2e) displayed (blue) for all four K80 amino sidechain groups in the tetramer. R311 and E302 lie outside of this contour, indicating low interaction with K80, consistent with pH-coupling. Other listed groups lie within the contour and therefore have potential to interact directly with the K80 pH sensor residue(s).

With respect to residues that couple to pH-sensing, the R311-E302 salt-bridge of Kir1.1 is a good example. The mutation R311Q gives a large shift in pH_50_ for channel opening [21]. The predicted effect of this mutation is shown in Fig 2A, where calculated pH-dependence of ionisable group energy, differenced between the partially open and closed forms, is displayed for wild-type and R311Q mutant Kir1.1 models. Looking first at the wild type model, the slope of the pH-dependence is consistent with the known opening at higher pH, since the partially open form becomes less stable relative to the closed form as the pH decreases i.e. the closed form will be preferred at lower pH. This form of plot is the essence of pH-sensitivity seen in the pKa and pH-dependence calculations. A non-zero gradient with pH implies a change with pH, and the degree of slope relates directly to the pH switch. In this case, the slope remains largely the same, but the plot is up-shifted, for the R311Q Kir1.1 model. According to the predictions, R311 and E302 interact in both partially open and closed forms, with no change in ionisation at neutral pH, leading to comparable slopes in Fig 2A. However, the strength of the salt-bridge is somewhat stronger in the partially open form than in the closed form, leading to the upward shift in the plot of open-closed energetics for the R311Q mutant. As a result, this salt-bridge is coupled to pH-dependence at physiological pH, although it does not itself constitute a part of the pH-sensor, as would be defined by ionisation changes at physiological pH. Since this study is designed to look for potential pH-sensing residues, only ionisable groups are returned, but in principle any interaction that changes between open and closed forms will contribute to the channel opening energetics, and mutation of the residues involved could shift the open/closed equilibrium and thus be coupled to pH-dependence, as in Fig 2A. We have focussed on the slopes of pH-dependence plots in Fig 2A, but it is noted that the absolute value of ionisable group energy difference (open—closed) is large and positive over this pH range. Presumably this effective destabilisation of open over charged forms is compensated by other interactions, not included in the current work. As a consequence, it would be expected that there is ample scope for perturbing the open/closed equilibrium with mutation throughout the molecule.

There is a further class of residues that interact with pH-sensing groups. These can directly influence the pKa and interactions around pH-sensors, and thus become a part of the pH-sensor themselves. In Fig 2B a single monomer of Kir1.1 is shown, but with a positive potential envelope (at a level of kT/2e) generated from all 4 ionised K80 groups in the tetramer. The locations of residues falling into the class of those that can interact with a pH sensor (K80) are drawn, along with R311-E302. Whereas R311-E302 lies outside of this potential envelope, and will interact only weakly directly with K80, the other residues could interact more strongly with K80. These intermediate category residues have the potential to integrate with the pH sensor, and mutation of each of them has been observed to alter pH-dependence of Kir 1.1 [3,20].

**Fig 3:**
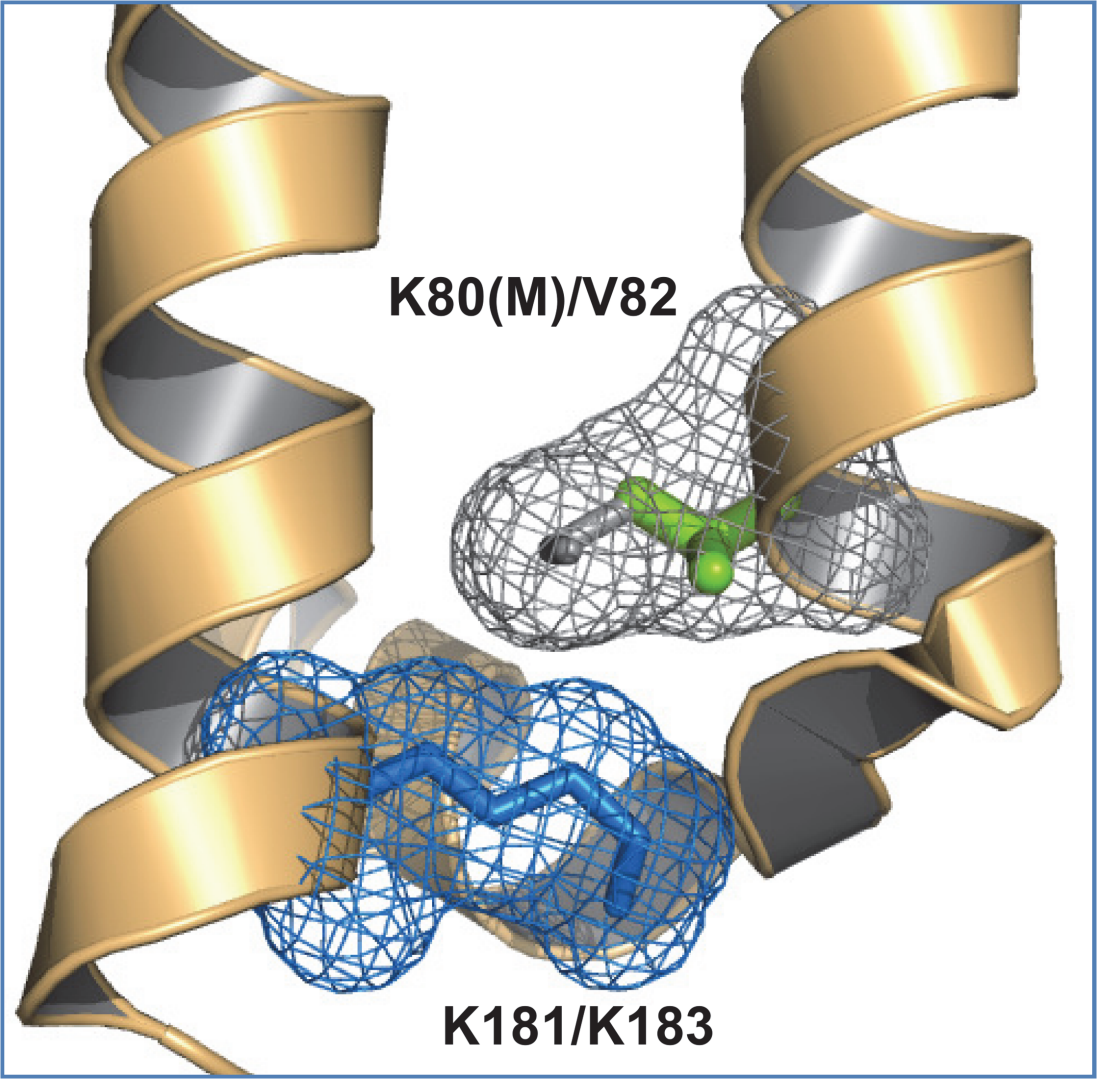
The environment of K181 in rat Kir1.1 compared with K183 of puffer fish Kir1.1. Whereas the key pH-sensing residue K80 (grey stick and mesh) is a neighbour of K181 (bue mesh and stick) in rat Kir1.1, the presence of valine (green stick) at the equivalent position in puffer fish could allow an altered conformation of K183 (not drawn).

Puffer fish Kir1.1 has valine in place of rat Kir1.1 K80, and adjacent to this site it has an additional lysine, also present in rat Kir1.1 (K181, Fig 3). The mesh surfaces in Fig 3 indicate that whereas K181 in rat Kir1.1 is restricted by the proximity of K80, in puffer fish Kir1.1 there would be flexibility for the amino group of the K181 sidechain (rat Kir1.1 numbering) to occupy a similar position to that of K80 in rat Kir1.1 This suggests that K181 in puffer fish Kir1.1 could play a pH-sensing role. It is known to be coupled to pH (although perhaps not pH-sensing as defined in the current work) in rat Kir1.1 [63].

Overall for rat Kir1.1, FD/DH calculations indicate that a relatively small subset of ionisable groups could be responsible for pH-sensing, smaller than would be derived from FDPB calculations. This conclusion though is in part dependent on the accuracy of using the modelled conformations for closed and indicative open forms. A much larger set of residues, ionisable and non-ionisable, will couple to pH-sensing.

### Prediction of pH-sensing residues in ASIC1a

**Fig 4:**
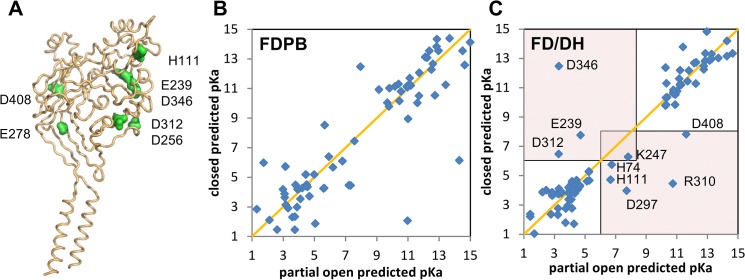
Prediction of pH-sensing residues for ASIC1a. (A) Cartoon of an ASIC1a monomer (from the trimer), with the transmembrane helices evident in the lower part, and residues found to influence pH-dependence (see text) marked as green surfaces in the extracellular region. (B) and (C) Scatter plots of predicted pKas for closed (desensitised) and partially open channels, with the orange diagonal indicating equal pKas in these forms. With a pH range of physiological interest between pH 6 and 8, the red shaded regions (panel C) contain groups for which ionisation is predicted to change between closed and partially open forms. Panel B is for FDPB calculations and panel C for FD/DH calculations, with various groups highlighted.

Table 1 and Fig 4A show CFRs identified in the literature. Fig 4B and 4C illustrate the distinction between FDPB and FD/DH calculations for the closed (desensitised) and partially open foms of ASIC1a, with the generally closer clustering to the diagonal for FD/DH indicative of ∆pKas that are closer to model compound values for FD/DH. If a scan for CFRs were being made with a single conformation and calculated pKas in the physiological range, fewer candidates would be generated with FD/DH, as the of majority acidic and basic residue predicted pKas would lie close to the model compound values (at acidic and alkaline pHs). Our strategy for CFR identification is concerned with the off-diagonal points in the FD/DH calculation particularly, with several of these labelled in Fig 4A. In S2 Fig panels A and B, the approximation of changing repacking tolerances in the FD/DH method, to mimic conformational change in partially buried residues, is shown. Of most interest here again are off-diagonal groups, those that alter ionisation state upon the packing changes. There is substantial overlap between the groups identified with the closed and partially open states, and their comparison with the results in Fig 4C is shown for the CFRs in Table 1. These groups are dominated by the carboxylic acids identified as potential sites of competition between protons and calcium [26]. Aspartic acid 356 stands out in Table 1, not being predicted as pH-sensing. This residue was identified as substantially altering pH_50_ upon mutagenesis [35]. Since D356 forms a salt-bridge with K212 from a neighbouring subunit in the 2qts structure, it is possible that pH_50_ alteration is mediated by a pH-coupling, rather than pH-sensing, mechanism. In that case, the pKas for D356 and K212 would not be in the physiological pH range, but the interaction between D356 and K212 would differ between open and closed forms. Inspection of the structures reveals that the distance between the D356 carboxylate and K212 amino groups almost doubles from a close salt-bridge in 2qts to a broken salt-bridge in 4fz0. These observations are consistent with the interpretation that the region around D356 is important in the pH-dependent transition of ASIC1a [35].

**Table 1:** Comparison of CFRs from predictions for ASIC1a and literature.

Amino Acid	Open—Closed	Closed (repacking)	Open (repacking)
**H111**	x	x	x
**E239**	x		x
**E278**		x	
**D312**	x	x	
**D346**	x	x	x
**D356**			
**D408**	x	x	x

Residues listed are those implicated in the literature as pH-sensing for ASIC1a (see text), followed by whether these amino acids are also highlighted in the three calculations (indicated by x). Open is the partially open 4fz0 structure and closed the 2qts desensitised structure. Description of the scheme that uses repacking to identify partially buried groups is given in Materials and Methods.

### pH-sensing residues and calcium-binding in ASIC1a

**Fig 5:**
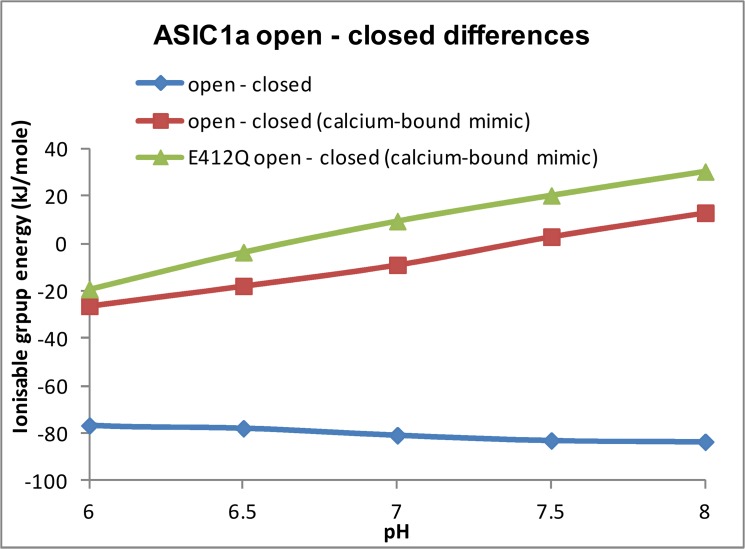
Calcium binding and pH-dependence in ASIC1a. Plots of predicted pH-dependence for the difference of partially open—closed (desensitised) channels are shown. The slope of a plot (blue) that takes no account of calcium binding is not in the right sense of relative open state stabilisation at lower pH, whilst that with carboxylic acid deletion to mimic calcium binding (red, see text) is consistent with channel opening. Finally, on the calcium-binding mimic background, the E412Q mutation (green) shifts the curve without substantially altering the slope.

Whilst the identification of possible calcium sensing groups comes directly from structure determination [26], and structure-based calculations also consistently identify them (Table 1), the detail of proton-calcium competition is much more complex to model directly and beyond the scope of the current empirical studies. It is possible however is to approximate calcium binding through mutation of the ionisable group charge. In our calculations, D346 and (partially) E239 are protonated in the closed, desensitised structure (2qts) of ASIC1a at pH 7, but both are predicted to be ionised in the toxin-bound open form (4fz0). So, although these residues are clearly CFRs from the calculations, the slope of pH-dependent stability change for the open to closed transition is unlikely to be correct, if calcium binds to the acidic groups when they are close to eachother in the closed form. Therefore, D346N and E239Q mutations are constructed in the closed form. Fig 5 shows the predicted pH-dependence of energy difference between open and closed forms for the cases of open—closed and open—closed/double charge mutant (as an approximation for calcium binding in the closed form). It is seen that the sense of pH-dependent stability change swaps over between these two difference calculations. A positive slope (as for the calcium-binding approximation) is the correct match to physiological behaviour, since this gives the open form becoming relatively more stable as pH falls through neutral pH. It is therefore possible to use these empirical calculations to identify CFRs and to match their influence to the sense of physiological pH-dependent channel opening or closing. In this case the pH-sensing residues are thought to be involved in proton competition with calcium.

### pH-coupled residues in ASIC1a

Although there are many groups that influence pH_50_ for channel opening [5,32,35], most are non-ionisable (which the current study is restricted to). One salt-bridge of interest is E412—R370, which does alter between closed and partially open states, but without predicted ionisation state change around neutral pH i.e. this would be pH-coupled rather than pH-sensing in our terminology. In order to study this salt-bridge further, it has been mutated (E412Q) in both closed and partially open forms, on the background of the calcium-bound closed form mimic in Fig 5. Thus Fig 5 also shows the pH-dependence of stability difference for modelled wild type (4fz0 – 2qts/D346N/E239Q) compared with that for E412Q mutant (4fz0/E412Q – 2qts/D346N/E239Q/E412Q). The slope around neutral pH is similar between the wild type and E412Q models, consistent with only a small role in pH-sensing. An overall shift between the plots is consistent with coupling to the pH-dependence, and is in the sense of relative destabilisation of the partially open form for the E412Q mutant. This agrees with a small observed reduction in pH_50_ for the equivalent mutation to E412Q (6.25 to 6.15) [5]. These changes in pH-dependence (both measured and calculated) are small, but demonstrate the case for pH-coupled sites in ASIC1a.

### pH-sensitive and pH-insensitive ASICs

**Fig 6:**
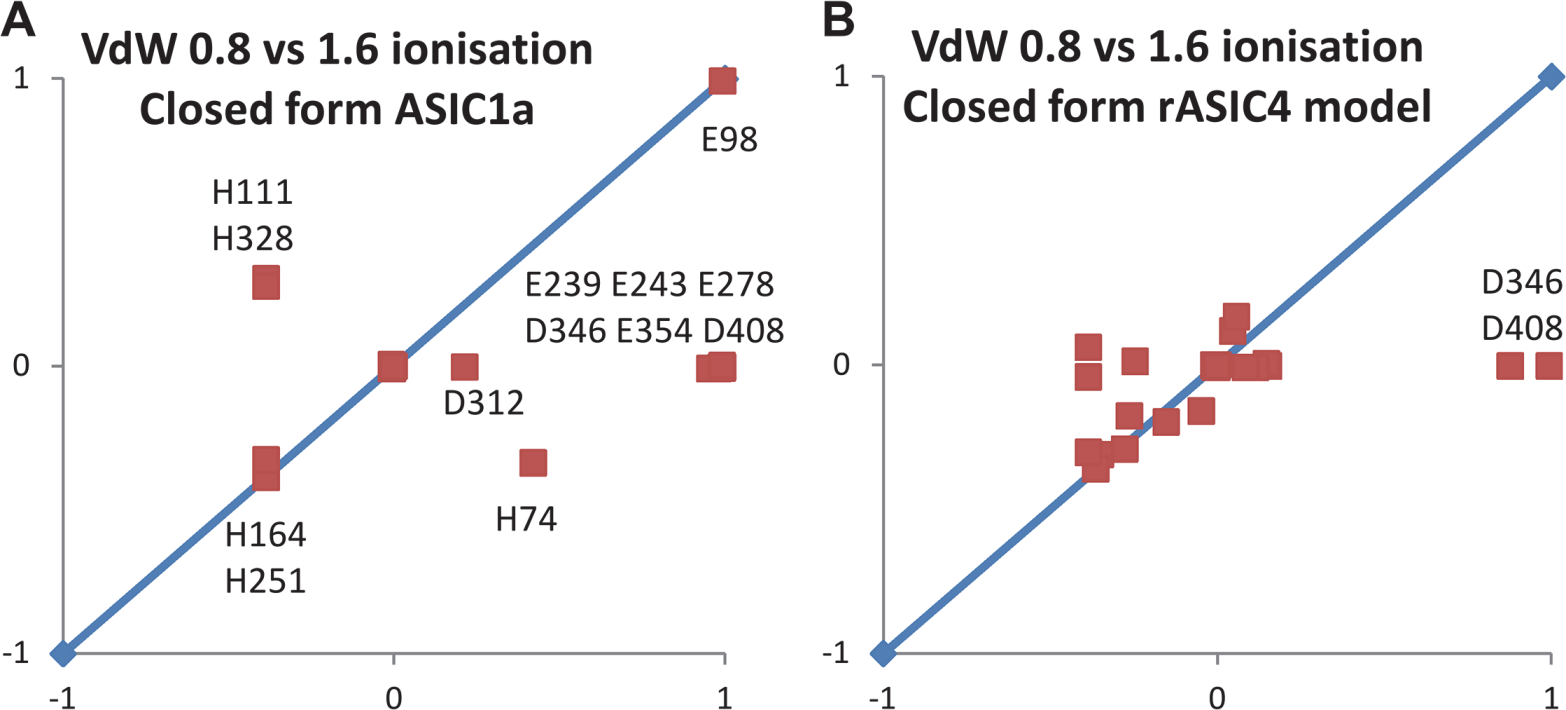
Predicted ionisation differences for packing changes of partially buried groups in pH-sensitive and pH-insensitive ASICs. (A) and (B) Scatter plots of ionisation changes (upon different repackings), calculated at pH 6.5, for the closed desensitised chicken ASIC1a structure (pH-sensitive, panel A), and a model of the equivalent structure for rat ASIC4 (pH-insensitive, panel B). Off-diagonal groups, those predicted to contribute to pH-dependence, are given with ASIC1a numbering.

ASIC1a (pH-sensitive) in the closed desensitised form was compared with a comparative model for rat ASIC4 (pH-insensitive). Solely the closed conformation was used, but with the repacking calculations to identify partially buried ionisable groups. It is the off-diagonal points in these plots that indicate groups predicted to change ionisation state at pH 6.5, with small conformational changes. There are many more off-diagonal groups for ASIC1a, (Fig 6A, which is replotted from S2A Fig here for clear comparison), than for rat ASIC4 (Fig 6B), consistent with this qualitative, empirical methodology giving insight to pH-dependence. Implicated groups for ASIC1a are dominated by the proposed calcium binding clusters of carboxylic acid containing amino acids [26]. There are many more of these groups in ASIC1a compared to ASIC4.

**Fig 7:**
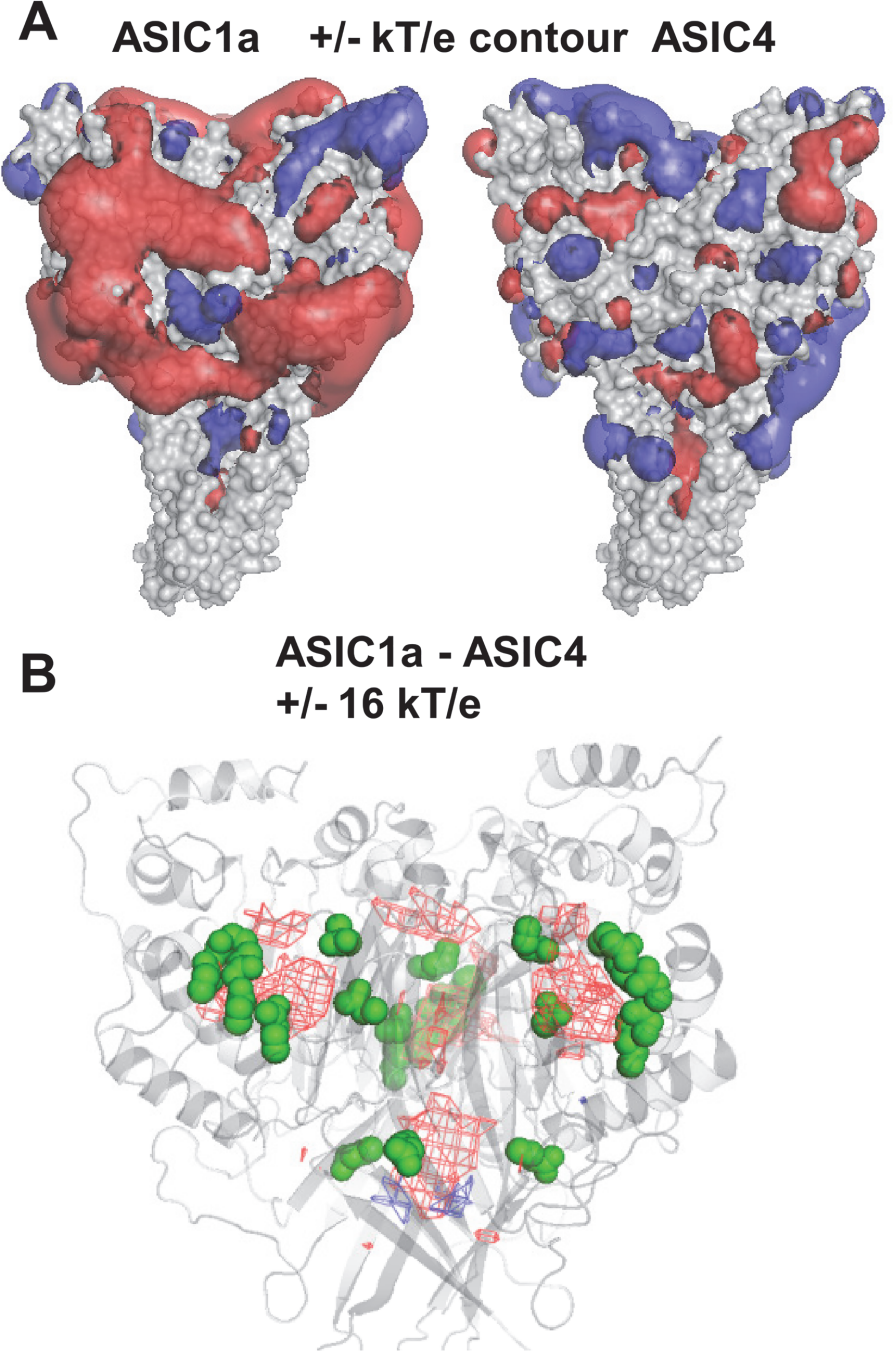
Charge differences between pH-sensitive and pH-insensitive ASICs. (A) Closed (desensitised) chicken ASIC1a structure and rat ASIC4 model, with electrostatic contours plotted (for physiological pH), at a relatively low contour (+/- kT/e, red is negative and blue shows positive potential). (B) The potential fields of (A) are differenced at a much higher level (+/- 16 kT/e) to identify the regions of most interest, which appear as the red mesh contour, indicating more negative contours in ASIC1a. These contours coincide with the groups indicated in the predictions of pH-sensitivity for ASIC1a (green surface) shown in Figs 4C and 6A. No calcium ions were included in these calculations.

In order to compare these pH-sensitive and pH-insensitive channels graphically, electrostatic potential maps have been plotted for each (Fig 7A). The difference in negative charges is readily apparent, with ASIC1a displaying a more negative surface than does ASIC4. A closer analysis of the distinction between these channels is made (Fig 7B) with a difference between the ASIC1a and ASIC4 maps, now contoured at a high level, and with the implicated groups from Fig 6A also plotted. Difference contours and the clusters of negatively charged amino acids overlap, emphasising the potential role for these amino acids in pH-sensitivity. This role was largely indicated in the original structural study [26]. The current work shows that for ASICs (as well as Kir channels), pKa calculations (particularly ones that avoid the over-prediction of large ∆pKas that is associated with single conformation FDPB methods), can focus searches for CFRs on to relatively small subsets of residues, reducing the demand on experimental resource for mutagenesis. In addition, it is possible to make predictions of residues which will be coupled to pH-dependence, without ionisation state changes in the physiological pH range, but at surfaces that change between conformational forms, and those that do alter ionisation state around gating pH and are genuine pH-sensors.

### Coupling to a pH transition

**Fig 8:**
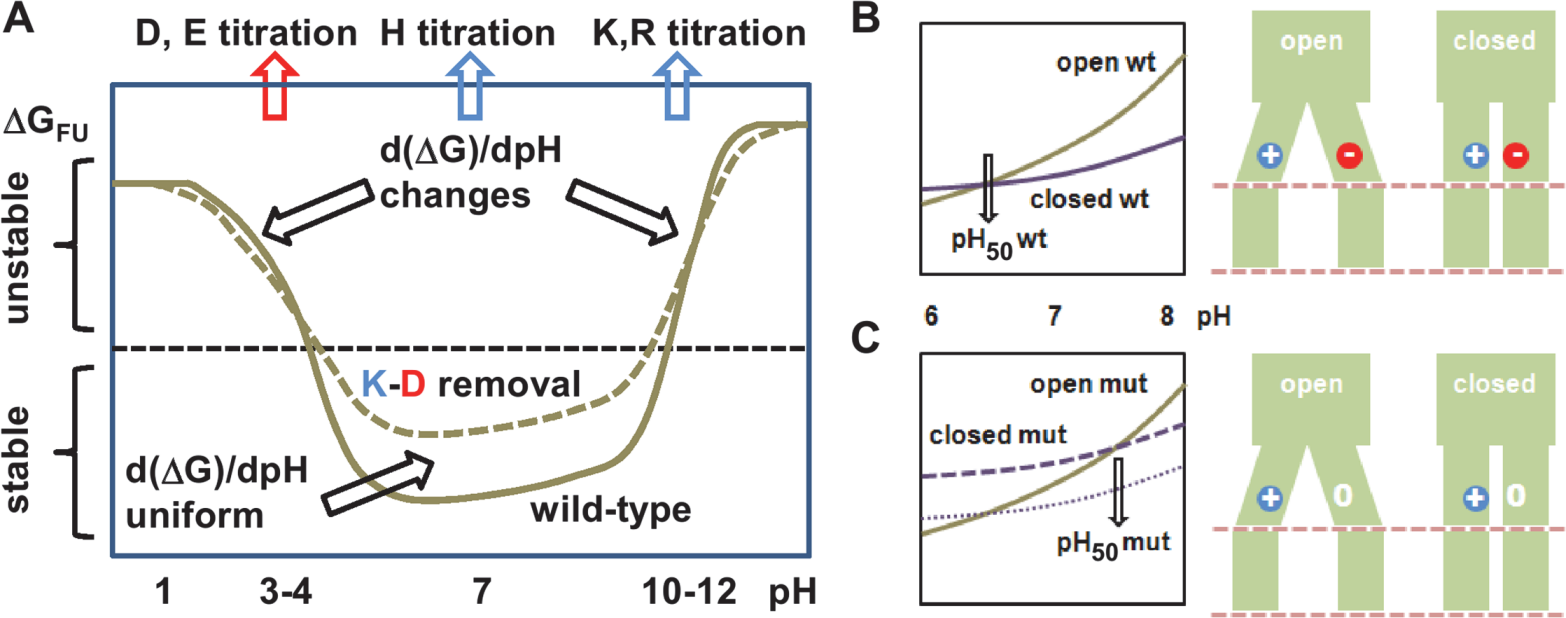
Schematic representation of coupling to a pH-dependent transition. In this example a salt-bridge is made in a channel closed form, but not in the open form, but this could be any type of interaction that varies between these forms. (A) General form of pH-dependent stability for a single folded conformation (F) relative to unfolded (U), ∆G_FU_. The standard pH titration ranges are marked for acidic and basic residues, omitting tyrosine, cysteine and amino acid termini, and ignoring groups with large ∆pKas. The effect of salt-bridge mutation has been exaggerated in scale, but importantly gives no change in slope of pH-dependence at neutral (physiological) pH. Wild type channel is shown schematically in panel B, together with schematic conformational energy curves for open and closed forms drawn such that they cross-over at pH_50_ around physiological pH. Upon salt-bridge mutation (loss of the acidic group, panel C), the open form curve remains the same as that for wild type, but the closed form is destabilised uniformly across physiological pH. As a result pH_50_ for the mutant form shifts, although there is no ionisation change at this pH between open and closed forms. The mutated interaction is therefore termed as coupled to pH-dependence, rather than underlying the pH-sensor itself.

It is instructive to look at a typical curve for the pH-dependence of protein stability (Fig 8A), generally with maximal stability at neutral pH where acidic and basic groups are ionised and interact, perhaps with salt-bridges. Removal of a salt-bridge (emphasised in Fig 8A) will reduce stability over the range in which the acidic and basic partners are ionised. This in turn gives changes in the slope of the pH-dependence plot around the ionisation ranges, but not at neutral pH (if the groups do not ionise around neutral pH). We now look at the situation in which such a salt-bridge is made in one conformational form (closed in this example) and unmade in another (open). Then, since the slopes of the pH-dependence plots and their open-closed form differences, do not differ at neutral pH, that salt-bridge itself is not a direct pH sensor. However (Fig 8B and 8C), since mutating the salt-bridge alters the stability of open versus closed forms at neutral pH, it will change the point at which the open and closed form pH-dependent stability plots crossover, and will therefore change the observed pH_50_ for channel opening. The residues involved in this salt-bridge are therefore coupled to pH-sensing, rather than themselves providing the pH sensor. The same argument relates to non-ionisable groups that alter their environment between open and closed forms and impact upon stability differences. Thus, many residues can, in principle, be identified as pH-coupled. The number of true pH-sensor residues, which contribute to changes (open versus closed forms) in the slope of the pH-dependence around physiological pH are likely to be much fewer. Experimentally though it can be hard to distinguish these classes of groups, since they both affect pH_50_. True pH-sensor residues should diminish the amplitude of a pH-dependent change, but this may be complicated by the involvement of other groups.

## Conclusions

Prediction and identification of CFRs for pH-dependent properties of ion channels has been problematic for various reasons, including availability of relevant conformational data, interpretation of whether residues that alter pH_50_ when mutated are genuinely pH-sensors, and computational methods that generally over-estimate the number of acidic and basic ionisable groups with sufficiently large ∆pKas to bring their pKas into the physiological pH range. This last topic is addressed in the current work with comparison of the FD/DH method to FDPB. It is possible, in principle, to alleviate the problem of large ∆pKas from FDPB by conformational sampling, e.g. from molecular dynamics, and coupling these to ionisation state changes, or with the MCCE method [50]. FD/DH methodology achieves a similar end by determining those groups which can become largely water accessible by sidechain rotamer changes, and including a Debye-Hückel scheme for such groups, thereby reducing the scale of interactions and ∆pKas. Differences between FDPB and FD/DH in the expected sense are evident (Fig 1B compared to Fig 1C and Fig 4B to 4C), with the consequence that predictions will give a reduced set of CFRs for experimental investigation. We hypothesised that partially buried ionisable groups could be over-represented amongst CFRs, since relatively small conformational changes will alter their charge interaction properties. This is borne out since variation of a parameter that controls FD/DH model response to burial gives similar CFRs to those from calculations that difference between open and closed conformations, and these groups are also implicated in the literature (Table 1). Thus, the FD/DH model also provides an empirical and approximate framework for assessing CFRs associated with pH-dependent conformational change, even when only a single conformation is available.

A close look at the form of pH-dependence allows distinction of true pH-sensing residues from those that are coupled to pH-sensors. Since pH-dependence associates with a slope in the plot of difference in conformational energy (between states) against pH, groups that contribute to that slope are pH-sensors. Such groups will have ionisation state changes between the conformational forms (e.g. open and closed), in the relevant pH range. Other groups can also alter the observed pH_50_ for a conformational transition, but can do this by altering the strength of interactions between the conformations (Fig 8). Such groups shift the pH-dependence plots but without changing their slope. We therefore term them pH-coupled as opposed to pH-sensor groups. Many of the residues implicated in experimental studies of pH-dependence (and which are not restricted to ionisable groups) are likely to be pH-coupled, rather than the pH-sensing residues of most interest. In practice there will be some crossover between pH-coupled and pH-sensor residues, particularly for groups neighbouring pH-sensors, and potentially with a direct influence on their pKas.

There will be ongoing developments in structure solution for channel open and closed forms. For example, another toxin-bound structure for ASIC1a has been recently reported to capture the open channel state [65], beyond the partially open state used in this work. Modelling presented here is largely empirical, but appears effective in correctly identifying CFRs for the Kir1.1 and ASIC1a test cases. Further work in this direction could be aimed at families of channels with pH-dependent and pH-independent members (at physiological pH), through large-scale comparative modelling. In the direction of more detailed modelling, there is also scope for inclusion of membrane components and ion binding (e.g. directly modelling the competition between protons and calcium ions in ASICs), as well as using new channel structures as further benchmarks.

## Supporting Information

S1 Fig Kir1.1 pH-dependence and sidechain burial. (A) and (B) Scatter plots of ionisable group ionisations, calculated at pH 6.5. Rather than closed and partially open forms, here the two different conformations in each panel are those with different sidechain repacking based on either closed or partially open, aimed at identifying partially buried groups (see Materials and Methods). Off-diagonal groups are denoted. (A) Closed form, (B) partially open form. Ionisation differences between charge in folded protein and charge of the isolated amino acid (sidechain) at pH 6.5 are plotted. With K and R sidechains positively charged at pH 6.5, the difference appears in the range from -1e to 0, depending on their charge in the protein, whilst D and E are in the range 0 to +1e.
(TIF)

S2 Fig ASIC1a pH-dependence and sidechain burial. (A) and (B) Scatter plots of ionisable group ionisations, calculated at pH 6.5. Rather than closed and partially open forms, here the two different conformations in each panel are those with different sidechain repacking based on either closed (desensitised) or partially open, aimed at identifying partially buried groups. Off-diagonal groups are denoted. (A) Closed (desensitised) form, (B) partially open form. Ionisation differences are plotted from the minimum (-1e) to maximum (+1e) values.
(TIF)
