# Supplementary figures and images for: Computational Tools for Interpreting Ion Channel pH-Dependence
Source: PLoS One. 2015 Apr 27;10(4):e0125293. doi: 10.1371/journal.pone.0125293 (PMC4411139; doi:10.1371/journal.pone.0125293)

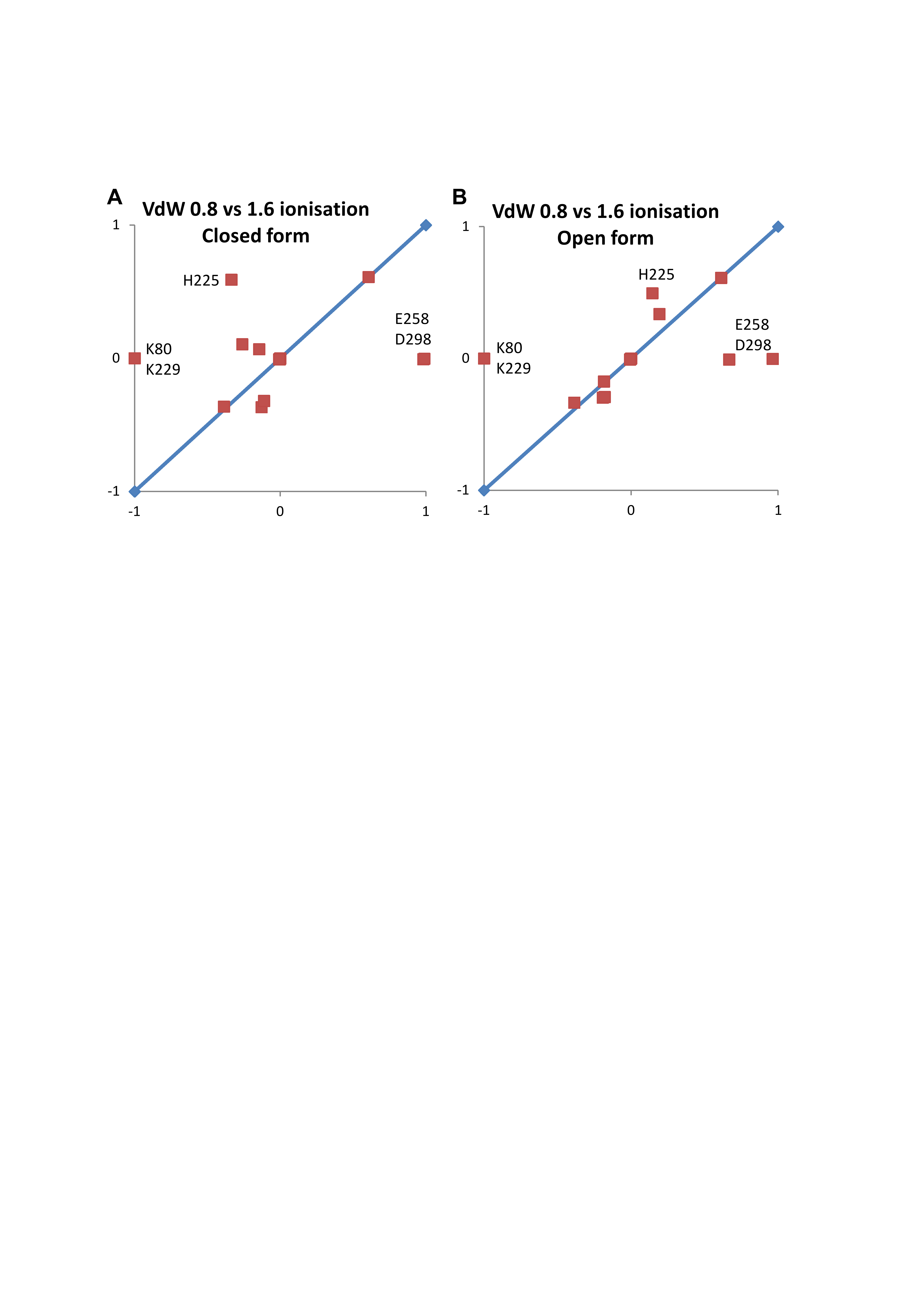

Supplement: S1 Fig — (A) and (B) Scatter plots of ionisable group ionisations, calculated at pH 6.5. Rather than closed and partially open forms, here the two different conformations in each panel are those with different sidechain repacking based on either closed or partially open, aimed at identifying partially buried groups (see Materials and Methods). Off-diagonal groups are denoted. (A) Closed form, (B) partially open form. Ionisation differences between charge in folded protein and charge of the isolated amino acid (sidechain) at pH 6.5 are plotted. With K and R sidechains positively charged at pH 6.5, the difference appears in the range from -1e to 0, depending on their charge in the protein, whilst D and E are in the range 0 to +1e. (TIF) [file pone.0125293.s001.tif]

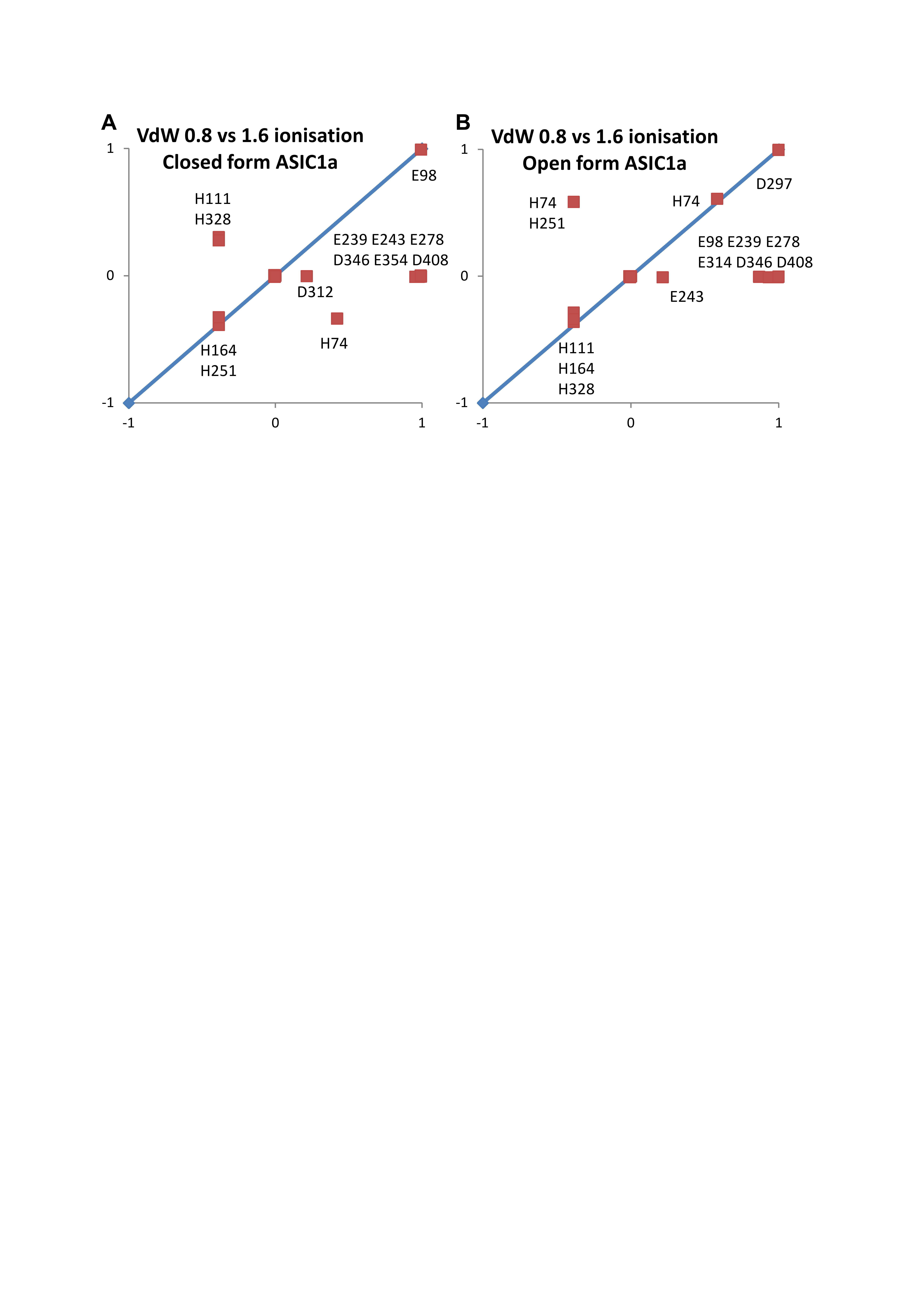

Supplement: S2 Fig — (A) and (B) Scatter plots of ionisable group ionisations, calculated at pH 6.5. Rather than closed and partially open forms, here the two different conformations in each panel are those with different sidechain repacking based on either closed (desensitised) or partially open, aimed at identifying partially buried groups. Off-diagonal groups are denoted. (A) Closed (desensitised) form, (B) partially open form. Ionisation differences are plotted from the minimum (-1e) to maximum (+1e) values. (TIF) [file pone.0125293.s002.tif]
